# Disruption of Phosphoinositide-Specific Phospholipases Cγ1 Contributes to Extracellular Matrix Synthesis of Human Osteoarthritis Chondrocytes

**DOI:** 10.3390/ijms150813236

**Published:** 2014-07-28

**Authors:** Guoqing Zeng, Xu Cui, Zejun Liu, Honghai Zhao, Xinpeng Zheng, Bing Zhang, Chun Xia

**Affiliations:** 1 Department of Sports Medicine & Joint Surgery, Zhongshan Hospital, Xiamen University, Fujian 361004, China; E-Mails: zenggqing@163.com (G.Z.); cuixu666@163.com (X.C.); 18850311056@163.com (Z.L.); 15960822127@163.com (H.Z.); zxp_635@126.com (X.Z.); 2 Medical School, Xiamen University, Fujian 361102, China

**Keywords:** PLC-γ1, human OA chondrocytes, ECM

## Abstract

Osteoarthritis (OA) is a degenerative joint disease characterized by articular cartilage degradation including extracellular matrix (ECM) degradation and cell loss. It is known that phosphoinositide-specific phospholipase γ1 (PLCγ1) can trigger several signaling pathways to regulate cell metabolism. However, whether this kinase is expressive and active in human OA chondrocytes and its role in the pathological progression of OA have not been investigated. The current study was designed to investigate the PLCγ1 expression in human OA cartilage, and whether PLCγ1 was involved in the ECM synthesis had been further explored using cultured human OA chondrocytes. Our results indicated that PLCγ1 was highly expressed in human OA chondrocytes. In our further study using the cultured human OA chondrocytes, the results demonstrated that the disruption of PLCγ1 by its inhibitor, U73122, and siRNA contributed to the ECM synthesis of human OA chondrocytes through regulating the expression of ECM-related signaling molecules, including MMP-13, Col II, TIMP1, Sox-9, and AGG. Furthermore, PLCγ1/IP3/Ca(2+)/CaMK II signaling axis regulated the ECM synthesis of human chondrocytes through triggering mTOR/P70S6K/S6 pathway. In summary, our results suggested that PLC-γ1 activities played an important role in the ECM synthesis of human OA chondrocytes, and may serve as a therapeutic target for treating OA.

## 1. Introduction

Osteoarthritis (OA) is a degenerative joint disease characterized by articular cartilage degradation including extracellular matrix (ECM) degradation and cell loss. The main components of ECM in cartilage are proteoglycans and collagenous network, and the loss of aggrecan (a negatively charged proteoglycan, AGG) and type II collagen (Col II) in ECM is the primary event leading to cartilage degradation [1]. It has been shown that collagens and aggrecan can be directly cleaved by matrix metalloproteinases (MMPs) and aggrecanases (ADAMTSs), both of which are regulated by some key signaling molecules. For example, MMP-2, MMP-9, and MMP-13 are activated by APC in human osteoarthritic cartilage chondrocytes [2]. Adaptor Proteins and Ras synergistically regulate IL-1-Induced ADAMTS-4 expression in human Chondrocytes [3]. However, the signaling regulatory mechanism is not fully elucidated.

Phosphoinositide-specific phospholipase γ (PLCγ), is one of key signaling molecules regulating cell metabolism [4,5]. Activation of PLCγ, which contains two isoforms (PLCγ1 and PLCγ2), can occur downstream of many receptor and non-receptor tyrosine kinases, and convert phosphatidylinositol 4,5-bisphosphate (PIP2) into the second messengers diacylglycerol (DAG) and inositol 1,4,5-trisphosphate (IP3) [4]. The former product activates protein kinase C isoforms, whereas the latter promotes the intracellular mobilization of Ca(2+) [4]. PLCγ isozymes are required for adhesion, spreading, and migration that are associated with ECM metabolism in a variety of cell types [5,6]. However, the role of PLCγ in ECM metabolism of chondrocytes, specifically, whether it is involved in the pathological progression of chondrocytes is less concerned, such as OA and RA.

In this study, we investigated the expression level of PLCγ in human normal and OA cartilage and cultured chondrocytes, and confirmed the involvement and regulatory mechanism of PLCγ1 in ECM metabolism of OA chondrocytes associated with mTOR/P70S6K/S6 pathway. The results from this project will provide additional evidence suggesting that PLC-γ1 activities play an important role in the ECM synthesis of human OA chondrocytes, and may serve as a therapeutic target for the prevention of OA.

## 2. Results

### 2.1. PLC-γ1 Is Highly Expressed in Human OA Chondrocytes

**Figure 1:**
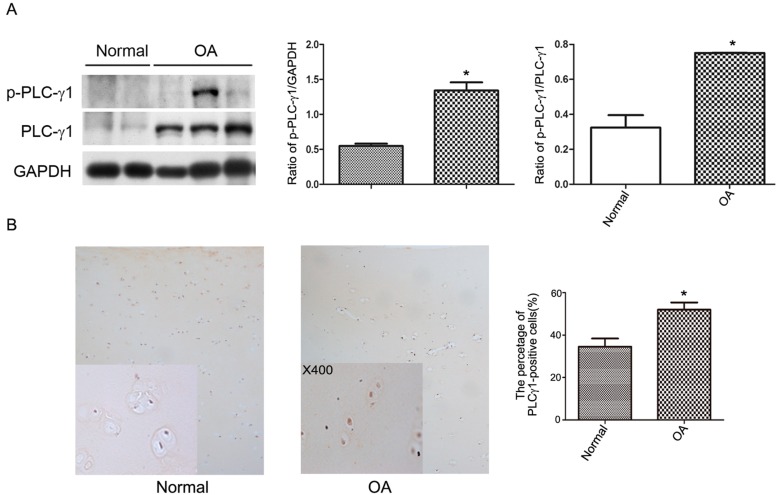
The expression level of phosphoinositide-specific phospholipase γ1 (PLCγ1) in normal articular and OA chondrocytes. Normal samples were obtained from 3 patients with amputation from accident, and OA samples were obtained from 20 patients with advanced OA. (**A**) The protein expression levels of PLCγ1 and p-PLCγ1 in cultured normal and OA chondrocytes were detected with western blotting analysis using rat anti-PLC-γ1, p-PLC-γ1, and GAPDH antibodies according to Materials and Methods. The values represent the mean ±S.E.M. of five independent experiments, each yielding similar results (* *p* < 0.05); (**B**) The protein expression level of PLCγ1 in normal articular and OA cartilage was detected with immunohistochemistry analysis according to Materials and Method (original magnification ×100 or ×400, * *p* < 0.05).

PLC-γ1 expression in cultured normal and OA chondrocytes obtained from human samples (Table 1) were detected with western blot analysis. The results depicted that the expression levels of PLCγ1 and p-PLCγ1-Tyr783, which is essential for PLCγ1 activation [1], were higher in human OA chondrocytes than normal chondrocytes (Figure 1A, * *p* < 0.05). Meanwhile, the expression level of PLC-γ1 in specimens of human normal and OA cartilage (Table 1) was monitored with immunohistochemistry technique. The results of Figure 1B showed that PLC-γ1 is highly expressed in OA cartilage, compared with normal cartilage (Figure 1B, * *p* < 0.05) indicating that PLC-γ1 has a higher expression in human OA chondrocytes.

**Table 1:** Information of osteoarthritis (OA) patients with total knee replacement surgery.

Age (Year)	Case	Sex		Duration of OA (Year)		* K.L.Image Criterion		Pro-Treatment Arthroscopy
		M	F	≤3	>3	III	IV	
60-	11	1	10	5	6	2	9	5
65-	15	5	10	4	11	4	11	2

* K.L Image criterion: Kellgren and Lawrecne criterion.

### 2.2. The ECM Synthesis of Human OA Chondrocytes Partly Depends on PLC-γ1

**Figure 2:**
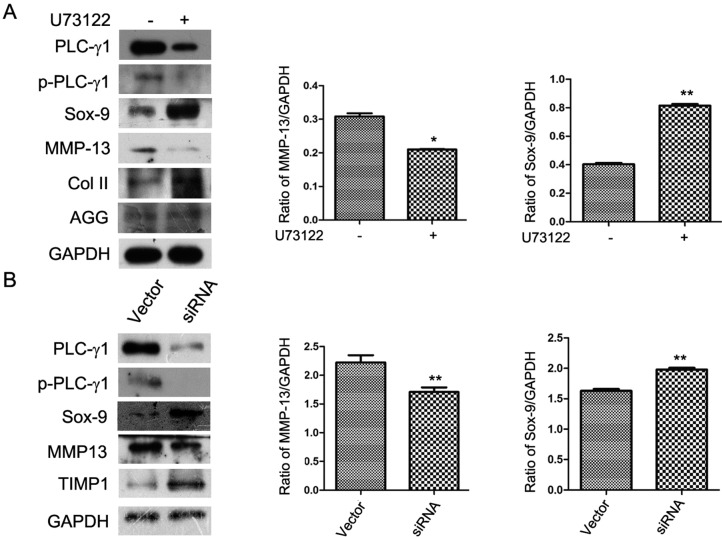
The extracellular matrix (ECM) synthesis of OA chondrocytes partially depends on PLC-γ1 activation. OA chondrocytes were obtained from 16 patients with advanced OA. (**A**) Cultured cells were treated with or without U73122 (5 μM) for 3 h, and the protein expression levels of PLC-γ1, p-PLC-γ1, Sox-9, MMP-13, Col II, and AGG were detected by western blotting analysis using rat anti-PLC-γ1, p-PLC-γ1, Sox-9, MMP-13, Col II, AGG, and GAPDH antibodies; (**B**) Cultured cells were transfected with or without siRNA-PLC-γ1, and the protein expression levels of PLC-γ1, p-PLC-γ1, Sox-9, MMP-13, and TIMP1 were detected by western blotting using rat anti-PLC-γ1, p-PLC-γ1, Sox-9, MMP-13, TIMP1, and GAPDH antibodies. The values represent the mean ±S.E.M. of three to five independent experiments, each yielding similar results (* *p* < 0.05, ** *p* < 0.01).

To investigate the role of PLC-γ1 in ECM metabolism of OA chondrocytes, cultured OA chondrocytes were treated with U73122 (PLC-γ1 specific inhibitor, inhibiting PLC-γ activity by decreasing the availability of PLC-γ substrate, PIP2 [7]) or transfected with PLC-γ1 siRNA (small interfering RNA). The expression levels of AGG, Col II, Sox-9, and MMP-13 were then detected using western blotting analysis. The expression levels of p-PLCγ1-Tyr783 and MMP-13 were reduced by the addition of U73122, while the expression levels of Sox-9, AGG, and Col II were enhanced (Figure 2A, * *p* < 0.05, ** *p* < 0.01). Similarly, the depletion of PLC-γ1 by siRNA enhanced Sox-9 and TIMP1 expressions, and reduced MMP-13 expression (Figure 2B, ** *p* < 0.01). Therefore, the blockade of PLC-γ1 could regulate the ECM synthesis, indicating that the ECM synthesis partly depends on PLC-γ1 activities.

### 2.3. PLC-γ1/IP3/Ca(2+)/CaMK II Axis Regulates the ECM Synthesis of Human OA Chondrocytes

**Figure 3:**
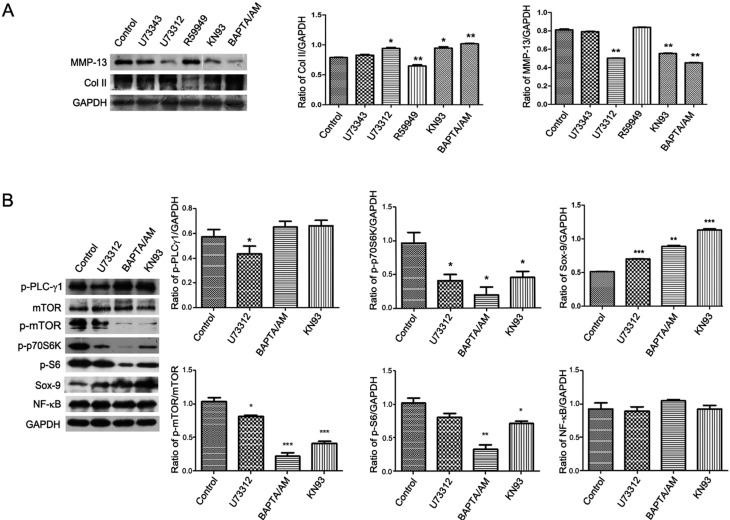
PLC-γ1/IP3/Ca(2+)/CaMK II axis regulates the ECM synthesis of human OA chondrocytes. OA chondrocytes were obtained from 16 patients with advanced OA. (**A**) Cultured cells were treated with or without U73122 (5 μM), U73343 (5 μM), R59949 (10 μM), KN93 (20 μM), and BAPTA/AM (10 μM) for 3 h, respectively. The protein expression levels of MMP-13 and Col II were detected by western blotting analysis using rat anti-MMP-13, Col II, and GAPDH antibodies; (**B**) Cultured cells were treated with or without U73122 (5 μM), KN93 (20 μM), and BAPTA/AM (10 μM) for 3 h, respectively. The proteinexpression levels of mTOR, p-mTOR, p-p70S6K, p-S6, Sox-9, and NF-κB p65 were detected by western blotting analysis using rat anti-mTOR, p-mTOR, p-p70S6K, p-S6, Sox-9, NF-κB p65, and GAPDH antibodies. The values represent the mean ± S.E.M. of three to five independent experiments, each yielding similar results (* *p* < 0.05, ** *p* <0.01, *** *p* <0.001).

Activated PLC-γ1 results in hydrolysis of PIP2 to DAG and IP3, which in turn leads to the activation of DAG/PKC and IP3/Ca(2+)/CaMK II axises [4]. The expression levels of MMP-13 and Col II in human OA chondrocytes were detected with western blotting analysis, when cells were exposed to different inhibitors, U73122, BAPTA/AM (the chelators of Ca(2+)), KN93 (CaMK II inhibitor), and R59949 (DAG-kinase inhibitor). The addition of BAPTA/AM and KN93 led to the decrease of MMP-13 expression and the increase of Col II expression in OA chondrocytes compared with untreated group, similar to the addition of U73122 (Figure 3A, * *p* < 0.05, ** *p* < 0.01). However, the addition of R59949 had no effect on MMP-13 and Col II expressions (Figure 3A). Based on the data, we detected the effect of U73122, KN93, and BAPTA/AM on those signaling molecules involved in regulating the ECM synthesis, including Sox-9, NF-κB (p65), mTOR, p70S6K, and S6 expression. The addition of U73122, BAPTA/AM, and KN93 reduced the phosphorylation levels of mTOR (p-mTOR), p70S6K (p-p70S6K), and S6 (p-S6), with the increase of Sox-9 expression and the alteration of NF-κB (p65) expression (Figure 3B, ** *p* < 0.01, *** *p* < 0.001). Therefore, the blockade of PLC-γ1/IP3/Ca(2+)/CamK axis up-regulated the ECM synthesis of human OA chondrocytes, associated with mTOR, p70S6K and S6 signaling molecules.

## 3. Discussion

In this study, our findings for the first time displayed the higher expression level of PLCγ1 in human OA chondrocytes, compared with human normal articular chondrocytes. The disruption of PLCγ1 activation by its inhibitor or siRNA changed the expression levels of ECM-related main signaling molecules, including MMP-13, TIMP1, Col II, Sox-9, and AGG. Furthermore, PLC-γ1/IP3/Ca(2+)/CamK II axis regulated the ECM synthesis of human OA chondrocytes through triggering the mTOR/P70S6K/S6 pathway. These data suggest that the ECM synthesis of human OA chondrocytes partly depends on PLCγ1, which may serve as novel drug target in treating OA.

Several lines of evidence supported that PLCγ is widely distributed, activated by receptor or non-receptor tyrosine kinases, and involved in cell proliferation, differentiation, apoptosis, and metastasis of mammal cells, including chondrocytes [4,5,6,8]. For example, Harada *et al.* [8] reported that PLCγ mediated FGFR3-induced STAT1 activation and this signaling cascade involved in the induction of apoptosis in the chondrogenic cell line ATDC5. Here, we demonstrated that PLCγ1 was expressed in normal articular and OA chondrocytes, and PLCγ1 was highly expressed in OA chondrocytes. These observations are consistent with Biswas’ report that PLCγ2 increased in the case of OA [9], suggesting that PLCγ1 could be involved in OA pathological progression.

The loss of AGG and Col II is the primary event of ECM degradation in OA progression [1], because they provide the compressive resistant and shock absorbing capability of cartilage under loading and tensile support for the tissue, respectively [10]. Meanwhile, MMP-13, which could cleave type II collagen, has been reported to express at high levels in OA cartilage [11]. Sox-9, a chondrogenic transcription factor that is responsible for matrix synthesis, had a significant decrease of gene and protein expression level in advanced OA cartilage [12,13]. Therefore, the variations of these signal molecules could serve as the markers for ECM degradation. Here, the increase of MMP-13 expression and the decrease of Sox-9 expression in OA chondrocytes had also been detected (Appendix 1A). In this study, we observed that the blockade of PLC-γ1 by U73122 and siRNA led to the increase of Col II, AGG, TIMP1, and Sox-9 expression, and the decrease of MMP-13 expression, indicating that the ECM synthesis of OA chondrocytes partially depended on PLC-γ1. It is consistent with previous studies that PLCγ1 regulated MMP3 expression in NIH 3T3 cells or aortic endothelial cells [14,15]. We thus suggest that the blockade of PLC-γ1 has chondroprotective effect by regulating ECM-related signaling molecules.

Several experimental data supported that intracellular Ca(2+) variations could mediate catabolic responses in OA chondrocytes [16,17]. In the adjuvant-induced arthritis rat model, treatment with amiloride, a voltage-gated Ca(2+) channel blocker, significantly decreased Mankin scores, while increased Col II and AGG mRNA and protein expression and reduced articular cartilage destruction [16]. More recently, it has been shown that intracellular calcium oscillations in articular chondrocytes induced by basic calcium phosphate crystals led to cartilage degradation [17]. This can be combined with our data that the PLC-γ1/IP3/Ca(2+)/CaMK II axis is more dominant than the PLC-γ1/DAG/PKC axis in PLC-γ1-dependent ECM synthesis of human OA chondrocytes. It is suggested that the increase of intracellular Ca(2+) induced by various stimulations might mainly regulate the ECM synthesis in OA progression.

In addition, the observation of the down regulation of PLC-γ1 by U73122 led to the dephosphorylation of mTOR, of p70S6K, and of S6 was intriguing, because the activation of mTOR could mediate coordinated changes in cell protein translation that are required for cell programs, including cell growth and apoptosis [18,19,20]. Presumably, PLC-γ might be associated with the regulation of mTOR/p70S6K/S6 pathway in human OA chondrocytes. It has been reported that PLC-γ could regulate mTOR/p70S6/S6 pathway in some cell lines. For example, Razmara *et al.* [21] reported that PLCγ was required for Akt activation (Ser473) that is responsible for the triggering of MTORC1/S6 pathway [22]. PLCγ contributed to PLD activation that was necessary for PDGF-BB-induced phosphorylation of S6 by mTORC1 in NIH3T3 cells [21]. PLC-γ was observed to drive the activation of mTOR/p70S6K pathway in Bcr-Abl-expressing cells [18]. Moreover, we very recently detected the effect of PLC-γ on mTOR expression in human OA chondrocytes (data not shown). Therefore, PLC-γ might play an important role in human OA chondrocytes via triggering the mTOR/p70S6K/S6 pathway. Meanwhile, similar to the participation of Ca-signaling, CaMK, and PKCs in the PLC-γ1/p70S6K/mTOR/S6 pathway by inhibiting these molecules in Bcr-Abl-expressing cells [18], our data of the down regulation of mTOR, of p70S6K, and of S6 by BAPTA/AM and KN93 demonstrated the involvement of the IP3/Ca(2+)/CaMK II axis in the PLC-γ1/p70S6K/mTOR/S6 pathway by inhibiting CaMK II and Ca(2+) in human OA chondrocytes. It is consistent with the report that the inhibition of Na/K-ATPase promoted myocardial TNF-α protein production and cardiac dysfunction during endotoxemia through activating Ca(2+)/CaMK/mTOR signaling [23]. Combined with our data that the ECM synthesis of OA chondrocytes partially depended on PLC-γ1 (Figure 1 and Figure 2) and the inhibition of rapamycin (mTOR inhibitor) enhanced the expression level of Col II in human OA chondrocytes (data not shown), we suggested that the PLCγ1/IP3/Ca(2+)/CaMK II signaling axis was involved in PLCγ1-depended ECM synthesis via triggering mTOR/P70S6K/S6 pathway.

## 4. Materials and Methods

### 4.1. Reagents and Antibodies

Antibodies against PLCγ1 (CST#2822S,), p-PLCγ1-Tyr783 (CST#2821S), mTOR (CST#2983), p-mTOR-Ser2448 (CST#2983), p-p70S6K-Thr389 (CST#9234S), p-S6-Ser235/236 (CST#2211S), GAPDH (CST#2251-1), NF-κBp65 (CST#8242), and PLCγ1 siRNA (CST#6293) were purchased from Cell Signaling Technology Inc. (Beverly, MA, USA). Antibodies against MMP-13 (SC-30073), Sox-9 (SC-20095), AGG (SC-16493), Col X (SC-323750), Col II (SC-52658), and PLCγ inhibitor (U73122), an inactive analog of U73122 (U73343), the chelators of Ca(2+) (BAPTA/AM), DG-kinase inhibitor (R59949), and Ca/Calmodulin-dependent-kinase (CaMK) inhibitor (KN93) were obtained from Sigma-Aldrich in China (Shanghai, China). Other reagents were of the highest grade commercially available.

### 4.2. Human Normal and OA Chondrocyte Isolation and Culture

Ethical approval for the study was obtained from the Ethics Committee of Zhongshan Hospital, Xiamen University (ID No. 20100426), China. After receiving all patient consent and in accordance with the hospital ethical guidelines, human normal cartilage was obtained from 3 patients (aged 35 years, 39 years and 42 years, 3 males) with amputation from accident, and OA cartilage was obtained from 26 patients (aged 60–72 years, 6 males and 20 females) with advanced OA who were undergoing total knee replacement surgery. The OA patients were diagnosed based on the criteria developed by the American College of Rheumatology Diagnostic Subcommittee for OA and had not taken any non-steroidal anti-inflammatory drugs or steroids for at least 2 weeks prior to surgery or had any intra-articular injection for at least 1 month prior to surgery. Articular cartilage was dissected from the femoral condyle and tibial plateau, one part was stored in liquid nitrogen for chondrocyte culture, another part was fixed in 4% paraformaldehyde for immunohistochemistry technique.

As described previously [24], the cartilage slices were minced and digested primarily with 1% trypsin for 0.5 h at 37 °C and subsequently with 0.2% (*v*/*v*) collagenase II for overnight in serum-free DMEM/F12 at 37 °C, collected by centrifugation (1000× *g* for 5 min) and washed twice with PBS. Then, cells were re-suspended and cultured in DMEM/F12 supplemented with 10% FBS (*v*/*v*) plus 1% penicillin/streptomycin. Within 2–3 days of harvesting, primary chondrocytes were cultured to 80% confluence and plated in 60-mm Petri dishes or 96-well plates. Prior to being used in the experiments, the expression of Col II (the main collagen type in ECM of chondrocytes) in chondrocytes was detected with immunohistochemistry technique (Appendix 1B). All of our clinical studies have been conducted according to the principles expressed in the Declaration of Helsinki.

### 4.3. Immunohistochemistry Technique

The sample was fixed in 4% paraformaldehyde for 48 h, decalcified in 15% EDTA (pH 7.0, 37 °C) for two weeks, and then paraffin-embedded for further routine histological preparation. As described in the manufacturer’s instructions (MAIXIN.BIO, Fuzhou, China), 3 μm tibia articular sections were incubated overnight at 4 °C with primary antibody: PLCγ1 (1:200 dilutions) and subsequently, with secondary antibody (1:400) for 60 mins. Diaminobenzidine (DAB) was used to visualize the immunohistochemical reaction followed by being counterstained with haematoxylin. Finally, dark brown cells were considered to be positive, and were counted throughout microscopically magnified fields (×400) of each articular cartilage section. The percentage of positive cells was analyzed by SPSS v.15.0 for Windows [25].

### 4.4. Protein Extraction and Western Blotting Analysis

Cells collected by centrifugation were lysed as previously described [26]. Protein extracts were electrophoresed on 8%–12% denaturing gel and transferred to PVDF membrane (GE Healthcare, Hertfordshire, UK) for western blotting analysis [26]. The signal was detected using a chemiluminescent detection system according to the manufacturer’s instructions (Pierce, Rockford, IL, USA).

### 4.5. Statistical Analysis

The differences between the groups were examined for statistical significance using Student’s *t*-test and one-way ANOVA with SPSS software. A value of *p* < 0.05 was considered as being significant.

## 5. Conclusions

**Scheme 1:**
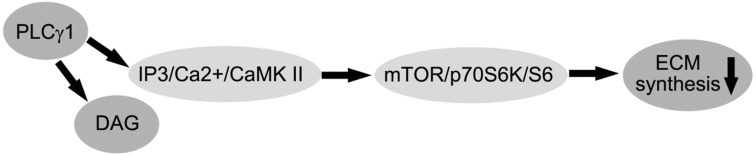
Schematic representation of regulatory mechanism of PLC-γ1 in the ECM synthesis of human OA chondrocytes.

In conclusion, PLC-γ1 is highly expressed in human OA cartilage, and involved in ECM synthesis of human OA chondrocytes. The disruption of PLC-γ1 pathway, in which the PLC-γ1/IP3/Ca(2+)/CamK axis was more dominant than PLC-γ1/DAG/PKC axis, contributes to ECM synthesis of human OA chondrocytes (Scheme 1). PLCγ1 may serve as a therapeutic target for the prevention of OA.

## Figures and Tables

**Appendix 1:**
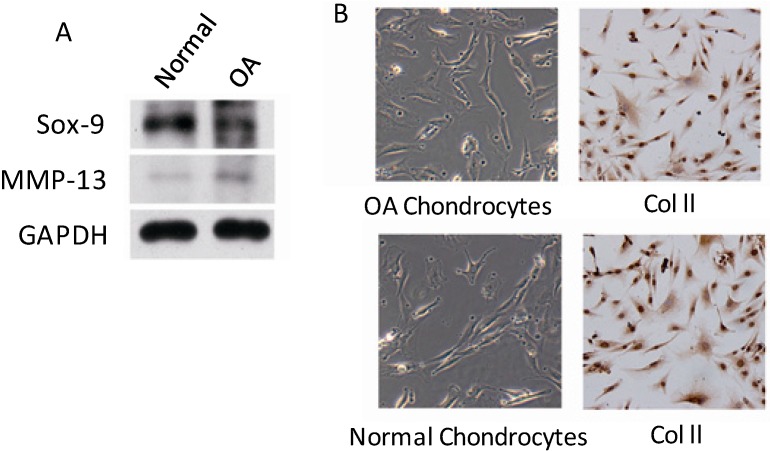
The expression of MMP-13, Sox-9, and Col II in normal and OA chondrocytes. (**A**) The protein expression levels of MMP-13 and Sox-9 in cultured normal and OA chondrocytes were detected with western blotting using rat anti-MMP-13, Sox-9, and GAPDH antibodies according to Materials and Methods; (**B**) Cultured chondrocytes were fixed in 4% paraformaldehyde for 20 min, and the expression of Col II was detected with immunohistochemistry analysis according to Materials and Method (original magnification ×100).
